# Establishment and characterization of a patient-derived circulating lung tumor cell line in vitro and in vivo

**DOI:** 10.1186/s12935-019-0735-z

**Published:** 2019-01-29

**Authors:** Zujun Que, Bin Luo, Zhiyi Zhou, Changsheng Dong, Yi Jiang, Lin Wang, Qihui Shi, Jianhui Tian

**Affiliations:** 1 Institute of Traditional Chinese Medicine Oncology, Shanghai Institute of Traditional Chinese Medicine, Shanghai, 200032 People’s Republic of China; 2 Department of Oncology, Longhua Hospital, Shanghai University of Traditional Chinese Medicine, Shanghai, 200032 People’s Republic of China; 3 Department of Nephrology, Longhua Hospital, Shanghai University of Traditional Chinese Medicine, Shanghai, 200032 People’s Republic of China; 4 Key Laboratory of Medical Epigenetics and Metabolism, Minhang Branch, Zhongshan Hospital and Institutes of Biomedical Sciences, Fudan University, Shanghai, 200433 People’s Republic of China

**Keywords:** Non-small cell lung cancer, Circulating tumor cell, Metastasis, Organ colonization, Prevention

## Abstract

**Background:**

Circulating tumor cells (CTCs) have been described as a population of cells that may seed metastasis, which is a reliable target for the prevention of metastases in lung cancer patients at the early stage. The culturing of CTCs in vitro can be used to study the mechanism of lung cancer metastasis and to screen antimetastasis drugs. This study aims to establish CTC cell line in vitro and explore the potential mechanism of its metastasis.

**Methods:**

A mixture of EpCAM- and EGFR-coated immunomagnetic microbeads in microfluidic Herringbone-Chip was used to capture CTCs. The CTCs, 95-D and A549 cells was evaluated by cell proliferation assays, clonal formation assays, migration assays and drug resistance. Flow cytometry and cytokine protein chip were used to detect the difference in phenotype and cytokine secretion between CTCs, 95-D and A549 cells. The NOD/SCID mice were used to study tumorigenicity, lung organ colonization and metastasis of CTCs. The H&E staining, immunohistochemistry and immunofluorescence assay were used to detect the pathological status of CTCs.

**Results:**

The number of EpCAM(+)/EGFR(+)/CK(+)/CD45(−) lung CTCs showed a weak negative correlation with clinical stages in patients with non-small cell lung cancer (NSCLC). In a phase IIa lung cancer patient, we successfully establish a permanent CTC cell line, named CTC-TJH-01. In vitro studies showed the CTC-TJH-01 cells were in the intermediate stage of epithelial to mesenchymal transition (EMT), had stem cell characteristics and were drug resistant. In vivo studies showed that CTC-TJH-01 cells can induce tumorigenesis, lung organ colonization and metastasis after xenografting in immunodeficient mice. In addition, the low expression level of CX3CL1 and high expression level of CXCL5 in the CTC-TJH-01 cells may be an important mechanism for their metastasis.

**Conclusions:**

We successfully established a permanent CTC cell line with metastatic ability, which can be used to screen antimetastatic drugs and study the mechanism of lung cancer metastasis.

## Background

Metastasis is the main leading cause of death in lung cancer, and currently lacking effective anti-metastatic drugs [1]. The main reason is that the existing treatments and drug development are based on the design of the primary tissues and cells of lung cancer, rather than the seeds of metastasis—circulating tumor cells (CTCs), which are the key cause of the poor curative effect [2]. Several large-scale clinical trials and meta-analyses have shown that the CTC count is an important indicator of the therapeutic effect, risk of progression and death in lung cancer patients [3, 4]. Cheng and his colleagues reported that the CTCs number appears to be significantly associated with bone metastasis from lung cancer [5]. In addition, the molecular characterization of single CTCs has revealed important information about the genotype and phenotype of these tumor cells, and has demonstrated their striking heterogeneity [6]. However, the current challenge is to analyze and identify subgroups of CTCs that can actually cause metastasis, and to design anti-metastatic drugs for this subgroup. Therefore, it is urgent to amplify CTC in vitro and establish a stable CTC cell line.

There has been an increasing effort to develop CTC capture methods, and the focus is now on expanding the quantity of CTCs using cell culture systems to provide sufficient cells for functional analyses. To the best of our knowledge, permanent CTC cell lines have only been reported for breast cancer, pancreatic cancer, small cell lung cancer and colon cancer [7–10]. Two other reports described a three-dimensional co-culture system for prostate cancer and early stage lung cancer CTCs in which the cells survived for only 14 days [11, 12]. The main reason is that the number of CTC in peripheral blood is very rare, and the condition of CTC culture in vitro is complicated. Despite this, we found that the establishment of functional CTC cell line models is now feasible.

If the CTC cell line can be successfully cultured and established in vitro, it will be used not only for single-cell sequencing, but also for surface marker detection, drug susceptibility testing, and construction of circulating tumor cell-derived xenograft (CDX) models. A recent study shown that exon allele mutations in prostate cancer CTCs were more frequent than in primary and metastatic tumors, and that CTCs were more heterogeneous [13]. Schölch also found that most colorectal cancer CTCs were dormant and had immune escape phenotype [14]. In addition, Yu et al. found that CTC cell lines from breast cancer patients have some tumorigenicity in immunodeficient mice, which also found that CTC cell lines were sensitive to paclitaxel and capecitabine, but resistant to fluvastatin, adriamycin and olaparib in drug sensitivity tests [7]. The above studies reveal the biological properties of CTC in various aspects.

In this study, we explore the relationship between the number of CTC and the clinical stage of NSCLC. Furthermore, we used a microfluidic chip combined with immunomagnetic separation technology to establish circulating tumor cell line from peripheral blood samples of NSCLC. We have systematically studied CTC-TJH-01, 95-D and A549 cells, and tried to elucidate the mechanism of CTCs metastasis.

## Methods

### Patients and CTCs analysis

This single institution prospective study was conducted at the Longhua Hospital (Shanghai, China). A total of 109 consecutive patients with pathologically confirmed NSCLC patients with stage I, II, III, or IV disease were enrolled into the study between November 2014 and January 2016. Patients were due to commence treatment with standard cytotoxic therapy. Those who underwent postsurgical chemotherapy, radiotherapy, or targeted therapy to the primary tumor or the sites of metastasis were permitted entry to the study after 8 weeks. Other inclusion criteria included World Health Organization Performance Status 0–2 and the ability to provide fully informed, written consent. Patients with a history of prior malignancy within 5 years of study entry were excluded. All patients gave written, informed consent and the study was approved by the ethics Committee of the Longhua Hospital.

Peripheral blood (5 ml) was collected from each patient into EDTA-containing blood collection tubes. A one-step method of CTCs detection was performed using a microfluidic Herringbone-Chip and immunomagnetic microbeads, as previously published [15]. Data on patient age, histological subtype, treatment received, clinical stage and the cell culture were collected. Isolated CTCs were cultured in non-adherent plate with a culture medium containing RPMI-1640 medium, epidermal growth factor (EGF), fibroblast growth factor 2 (FGF2) and B27 supplement. The detailed procedure can be found in our previous study [15].

### CTC culture

CTC-TJH-01 cells were obtained from the peripheral blood of patients with Stage IIa female lung adenocarcinoma after operation. A549 and 95-D human lung cancer cell lines were obtained from the Cell Bank of the Chinese Academy of Sciences (Shanghai, China). CTC-TJH-01 and A549 cells were grown in F12K medium containing 10% FBS and penicillin–streptomycin (Gbico Life Technologies, Carsbad, CA, USA). 95-D cells were grown in RPMI-1640 medium (Corning, Shanghai, China) containing 10% FBS and penicillin–streptomycin. All cells were grown at 37 °C in a humidified atmosphere with 5% CO_2_.

### Animals

Male NOD/SCID and C57BL/6 mice were born and housed in Shanghai Biomodel Organism Science & Technology Development under pathogen-free conditions in accordance with the Guide for the Care and Use of Laboratory Animals. All procedures were approved by the Animal Research Committee of Longhua Hospital Shanghai University of Traditional Chinese Medicine.

### Morphological observation

A Leica DMI3000B inverted microscope (Wetzler, Germany) and a DFC310FX Digital Camera were used for capturing images of the CTC-TJH-01, 95-D, and A549 cells.

### In vitro cell growth assays

CTC-TJH-01, 95-D, and A549 cells were plated at a density of 1 × 10^4^ cells/ml in 200 μl/well culture medium in 96-well plates. Cells were counted every 24 h using a cell counting kit-8 (CCK-8) assay (Dojindo) and growth curves were prepared.

### Colony formation assay

CTC-TJH-01, 95-D, and A549 cells (500 cells/well) were seeded into 6-well dishes and grown for 10 days in complete medium. Colonies were fixed in 4% paraformaldehyde and then stained with Giemsa. Plated were scanned, and colonies were counted.

### Cell migration assay

Quantitative cell migration assays were performed as described previously [16]. Briefly, the lower chamber was filled with 600 μl culture medium containing 30% FBS, and 1 × 10^6^ cells/ml in 100 μl serum-free medium were added into the upper chamber. Cells were allowed to migrate for 12 h at 37 °C. The remaining cells were then removed from the upper membrane surface by scraping with a cotton swab, and migrating cells were fixed with methanol, stained with Giemsa, and then photographed using an inverted microscope. The migration rate was assessed by counting the number of stained cells from 10 random fields at 200× magnification.

### Drug sensitivity assay

CTC-TJH-01, 95-D, and A549 cells were plated at a density of 2 × 10^4^ cells/ml in 200 μl/well culture medium in 96-well plates. After 24 h, the cells were treated with taxotere and cisplatin for 48 h, and cell viability was assessed using a CCK-8 assay.

### Flow cytometry assay

Flow cytometric analysis of surface phenotypic markers and nuclear factor for CTC-TJH-01, 95-D, and A549 cells was performed as per the manufacturer’s recommendation. Cells were collected and stained with different antihuman antibodies for 60 min, and combined with the corresponding fluorescence secondary antibody. The stained cells were analyzed by Cell Quest software on a FACScan flow cytometer (BD Biosciences, CA). Antibodies against E-cadherin, N-cadherin, Twist and Snai1 were purchased from Proteintech. Antibodies against CD47, CK-7, ALDH1 and CD45 were purchased from Abcam. Antibodies against Sox-2, PD-L1 and CD44 were purchased from cell signaling Technology (Cell signaling, US). An antibody against CD133 was obtained from Miltenyi.

### Cytokine chip assay

To study the underlying mechanism of metastasis in CTC-TJH-01 cells, we used a human cytokine antibody array (AAH-CYT-1000, RayBiotech, Inc.) to detect cytokine secretion in CTC-TJH-01, A549, and 95-D cell culture supernatants. This work was completed by Shanghai Yingbio Technology, Co., Ltd.

### RNA interference

RNA interference assays were performed as described previously [17]. Cells were transfected with CXCL5 siRNA (sih-CXCL5_001: 5′-CGTTGCGTTTGTTTACAGA-3′, si-h-CXCL5_002: 5′-GCAAGGAGTTCATCCCAAA-3′, si-h-CXCL5_003: 5′-GGAAGGAAATTTGTCTTGA-3′) when they reached 60% confluence, using ribo*FECT*™ CP Transfection Kit (stQ0007804-1, RiboBio, China). An unrelated, scrambled siRNA was used as negative control.

### Peripheral blood mononuclear cells isolate and co-culture

PBMC was extracted from peripheral blood (2 ml) by Ficoll density gradient centrifugation. PBMC and lung cancer cells were co-culture in transwell chamber at the ratio of 20:1 for 4 h, which allows PBMC to move from the upper chamber to the lower chamber. Then the PBMC of the lower chamber was collected and stained with fluorescent label antibody, and detected by flow cytometer. Fluorescent label antibodies FITC-CD3, PerCP-Cy5.5-CD4, and PE-CD8 were purchased from BioLegend.

### Tumor growth and lung metastasis assays

CTC-TJH-01 cells and A549 cells at a density of 1 × 10^7^ cells/ml in 100 μl were injected subcutaneously at the right abdomen of 6-week-old male NOD/SCID mice. Each group had 8 mice. Tumor development and growth were monitored twice a week, and tumor volumes were calculated using the formula [sagittal dimension (mm) × cross dimension (mm)^2^]/2 and expressed in mm^3^. After 5 weeks of inoculation, the mice were sacrificed, and the tumor was sectioned and stained by H&E and immunofluorescence.

For the lung organ colonization experiments, male C57BL/6 mice were intravenously injected with CTC-TJH-01 or A549 cells at a density of 1 × 10^7^ cells/ml in 100 μl. Every 2 weeks, two mice were sacrificed, and the lungs were removed. Lung was observed under an anatomical microscope, and detected by H&E and immunofluorescence. In addition, we also used NOD/SCID mice to inoculate CTC cells through the tail vein or subcutaneously, and then observed the lung metastasis.

### Immunohistochemistry and immunofluorescence assays

The subcutaneous tumors and lungs were fixed in 4% paraformaldehyde and embedded in paraffin blocks. The lungs and subcutaneous tumor sections were stained with H&E. The subcutaneous tumor sections were stained with various antihuman antibodies (CK-7, EGFR and K-ras). Staining was performed using a NEXES immunohistochemistry robot. Slides were scanned at 100× and 400× magnifications using a Leica TCS-SP8 laser confocal microscope.

### Statistical analysis

Data analysis was performed using SPSS software version 23.0 for Windows (IBM, USA) and Prism 5 (GraphPad Software, San Diego, CA, USA). Student’s t-tests were used to evaluate the significance of differences between experimental groups. Data are expressed as the mean ± SD. The levels of statistical significance were set at: *P < 0.05, **P < 0.01, and ***P < 0.001. All data points represent the mean of triplicates.

## Results

### CTCs count correlates negatively with clinical stage

A total of 98 consecutive patients with NSCLC were enrolled into the study between November 2014 and January 2016 (NCT 02603003). Eight patients were not eligible for CTC analysis because of insufficient blood volume available, and a history of ovarian carcinoma, which leaving 89 patients for inclusion. Patient demographics are listed in Table 1. Spearman analysis was used to identify associations between CTCs count and clinical stage in patients with NSCLC. The number of EpCAM(+)/EGFR(+)/CK(+)/CD45(−) lung CTCs showed a weak negative correlation with clinical stages in patients with NSCLC (r_s_ = − 0.258, *P *= 0.015; Table 2).

**Table 1:** CTCs counts, isolation and ex vivo expansion

Patient ID	CTC detection by one-step microfluidics-based immunomagnetic isolation		Ex vivo CTC culture
*N* = 89	*N* = 4	*N *= 85	*N* = 2
1		22	
2		3	
3		81	
4		49	
5		1	
6		28	
7		147	
8		10	
9		2	
10		22	
11		8	
12		1	
13		143	
14		11	
15		39	
16		15	
17		6	
18		22	
19		21	
20		33	
21		4	
22		3	
23		212	Yes
24		18	
25		9	
26		5	
27		7	
28		4	
29		227	
30		57	
31	0		
32		13	
33		1	
34		25	
35	0		
36		139	
37		12	
38		1	
39		3	
40		8	
41		26	
42		5	
43		6	
44		2	
45		3	
46		130	Yes^a^
47		5	
48		18	
49		16	
50		6	
51		187	
52		5	
53		11	
54		102	
55		177	
56		17	
57		72	
58		11	
59		18	
60		5	
61		9	
62		2	
63		9	
64		8	
65		27	
66	0		
67		65	
68		8	
69		10	
70		15	
71		17	
72		12	
73	0		
74		16	
75		1	
76		6	
77		279	
78		7	
79		3	
80		7	
81		7	
82		1	
83		3	
84		12	
85		28	
86		10	
87		2	
88		9	
89		4	

^a^From the female patients with IIA stage, we were able to expand CTCs in cell culture

**Table 2:** Analysis of the correlation between CTCs counts with NSCLC clinical stage

Stage	No. of cases (n)	CTC count (mean ± SD)	95% CI	r_s_	P-value
I	26	49.46 ± 13.34	26.04–80.81	− 0.25	0.015
II	17	23.53 ± 13.32	5.28–52.53		
III	16	29.88 ± 13.66	6.29–58.37		
IV	30	14.63 ± 4.38	8.42–23.50		

*CTC* circulating tumor cells

### Ex vivo expansion of CTCs has strong drug resistance and metastatic ability

We isolated the CTCs and performed ex vivo culture, and 2 of them (~ 2.2%) showed successful ex vivo CTC expansion. Long-term CTC cultures (> 6 months) were finally established from 1 (~ 1.1%) lung adenocarcinoma patient (a Stage IIa patient), and this example of CTCs was named CTC-TJH-01 cells.

**Fig. 1:**
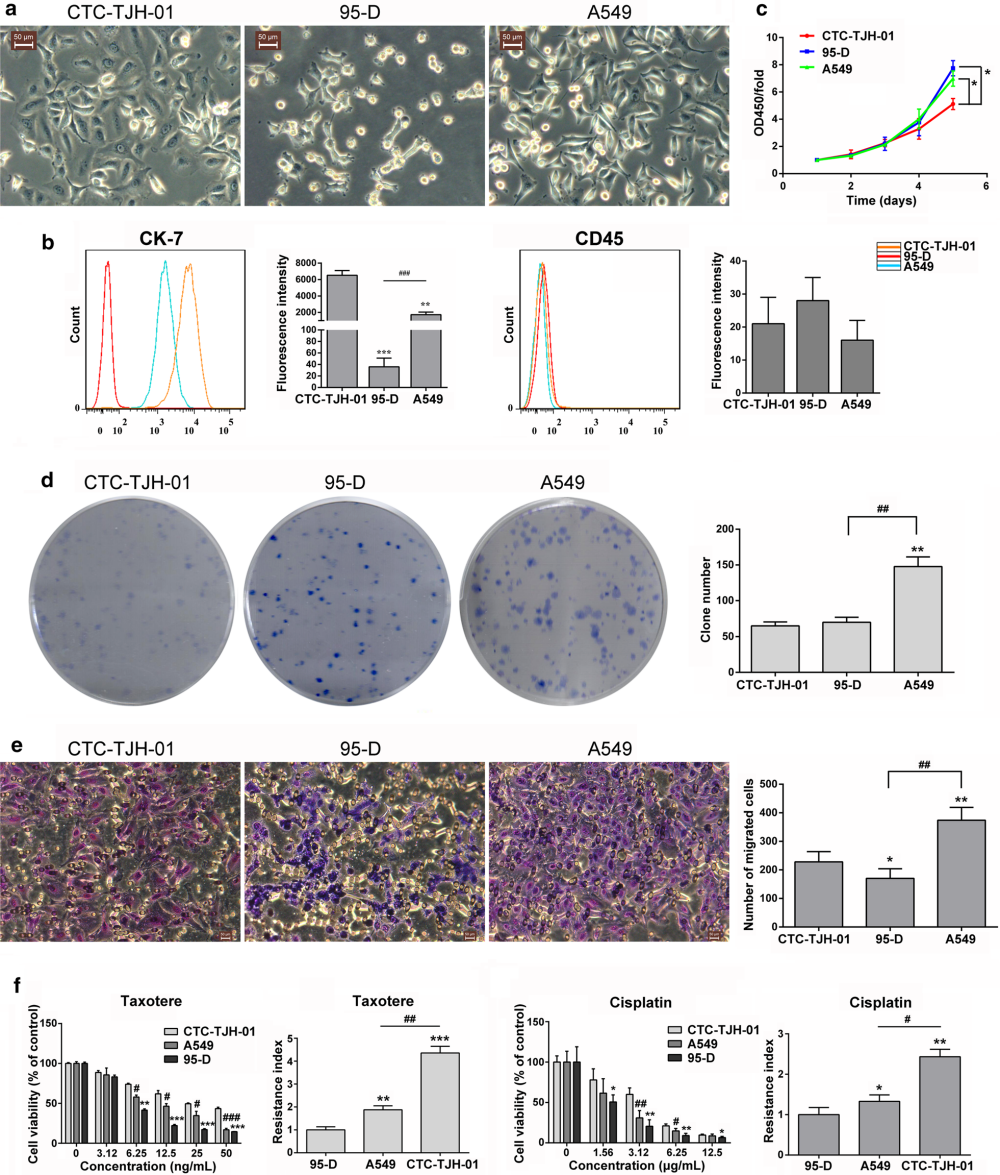
Distinct cell biological characteristics of CTCs. **a** Morphological observation of the CTC-TJH-01, 95-D and A549 cells under an inverted microscope. Scale bar, 50 μm. **b** Phenotype detection of CTC-TJH-01, 95-D and A549 cells. **c** Growth curve analyses of the CTC-TJH-01, 95-D and A549 cells. **d** Colony formation ability analyses of the CTC-TJH-01, 95-D and A549 cells. **e** Comparison of the transfer ability of the CTC-TJH-01, 95-D and A549 cells. **f** Comparison of the drug sensitivity of the CTC-TJH-01, 95-D and A549 cells to taxotere and cisplatin. Each bar represents the mean ± SD of three separate experiments. *P < 0.05; **P < 0.01; ***P < 0.001

In vitro study found that the CTC-TJH-01 cells had blebbing surfaces, prominent nucleoli and high nucleus-to-cytoplasm ratios, which were significantly larger than both the A549 cells and 95-D cells (Fig. 1a). In addition, we found that CTC-TJH-01 cells highly express CK-7 protein (Fig. 1b). When compared with the A549 cells and 95-D cells, the CTC-TJH-01 cells have weaker ability to proliferation, colony formation and metastasize, but it is more resistant to cisplatin and taxotere (Fig. 1c–f). These results indicate that the proliferation and metastasis ability of CTC-TJH-01 cells is weak, but the drug resistance is stronger.

### CTC-TJH-01 cells are shown an intermediate epithelial/mesenchymal phenotype, stem cell-like characteristics, and immune escape characteristics

**Fig. 2:**
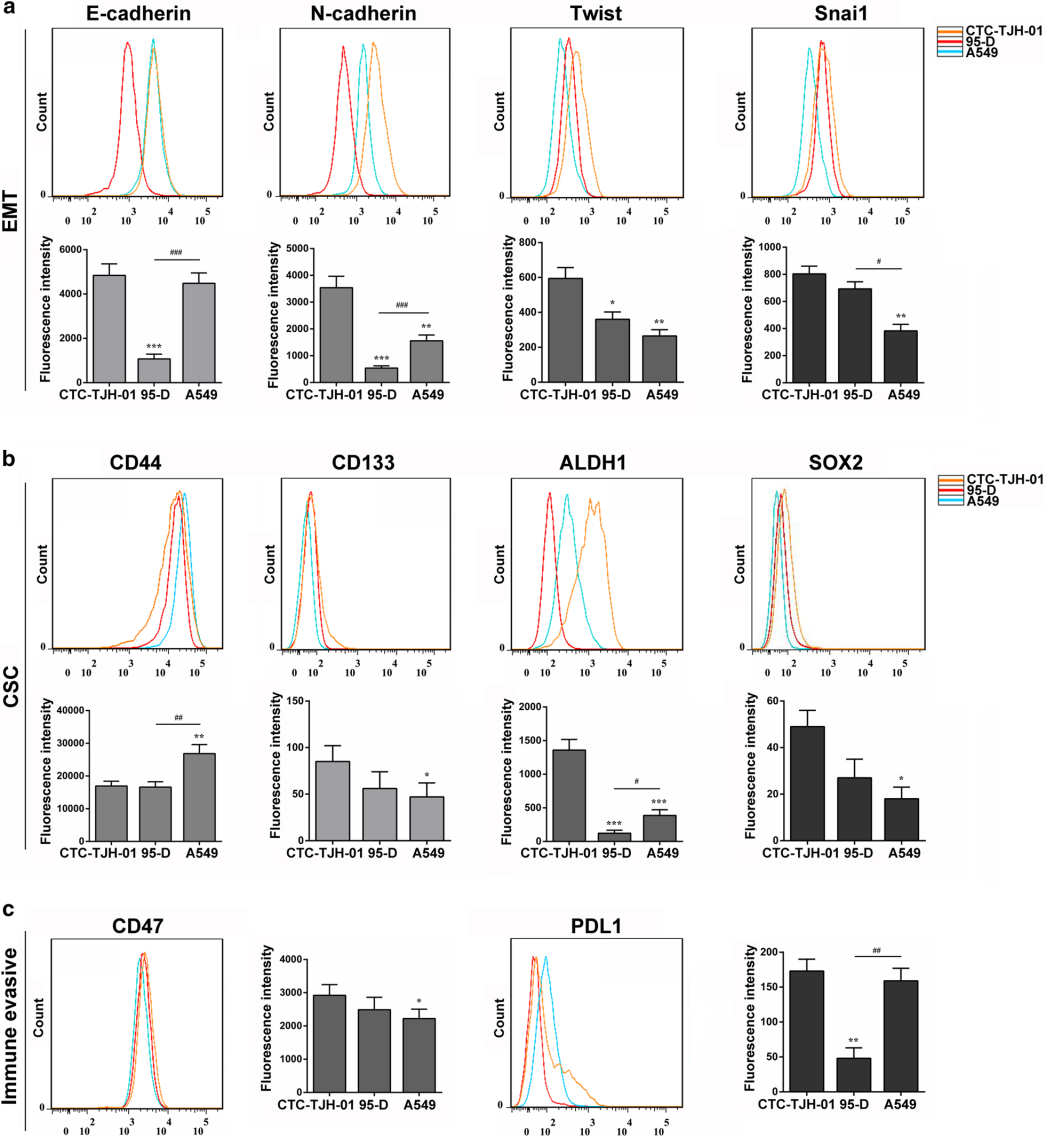
Altered immunological features of CTCs. **a** Comparison of EMT related protein expression in CTC-TJH-01, 95-D and A549 cells. **b** Comparison of lung cancer stem cells related protein expression in CTC-TJH-01, 95-D and A549 cells. **c** Comparison of immune escape related protein expression in CTC-TJH-01, 95-D and A549 cells. Each bar represents the mean ± SD of three separate experiments. *P < 0.05; **P < 0.01; ***P < 0.001

To study the unique phenotype of the CTC-TJH-01 cell line, we compared it with 95-D and A549 cells. Phenotypic analysis shown that the CTC-TJH-01 cells highly expressed E-cadherin, N-cadherin, CD44, ALDH1, CD47 proteins, had low expression levels of Twist, Snai1, PD-L1 proteins, and had a low expression level or no expression of CD133 and Sox2 proteins (Fig. 2). The results showed that the CTC-TJH-01 cells were in the intermediate stage of EMT transformation, with stem cell phenotype and immune escape characteristics.

### The high expression of CXCL5 protein and low expression of CX3CL1 protein in CTC-TJH-01 cells, which may be the mechanism of metastasis

**Fig. 3:**
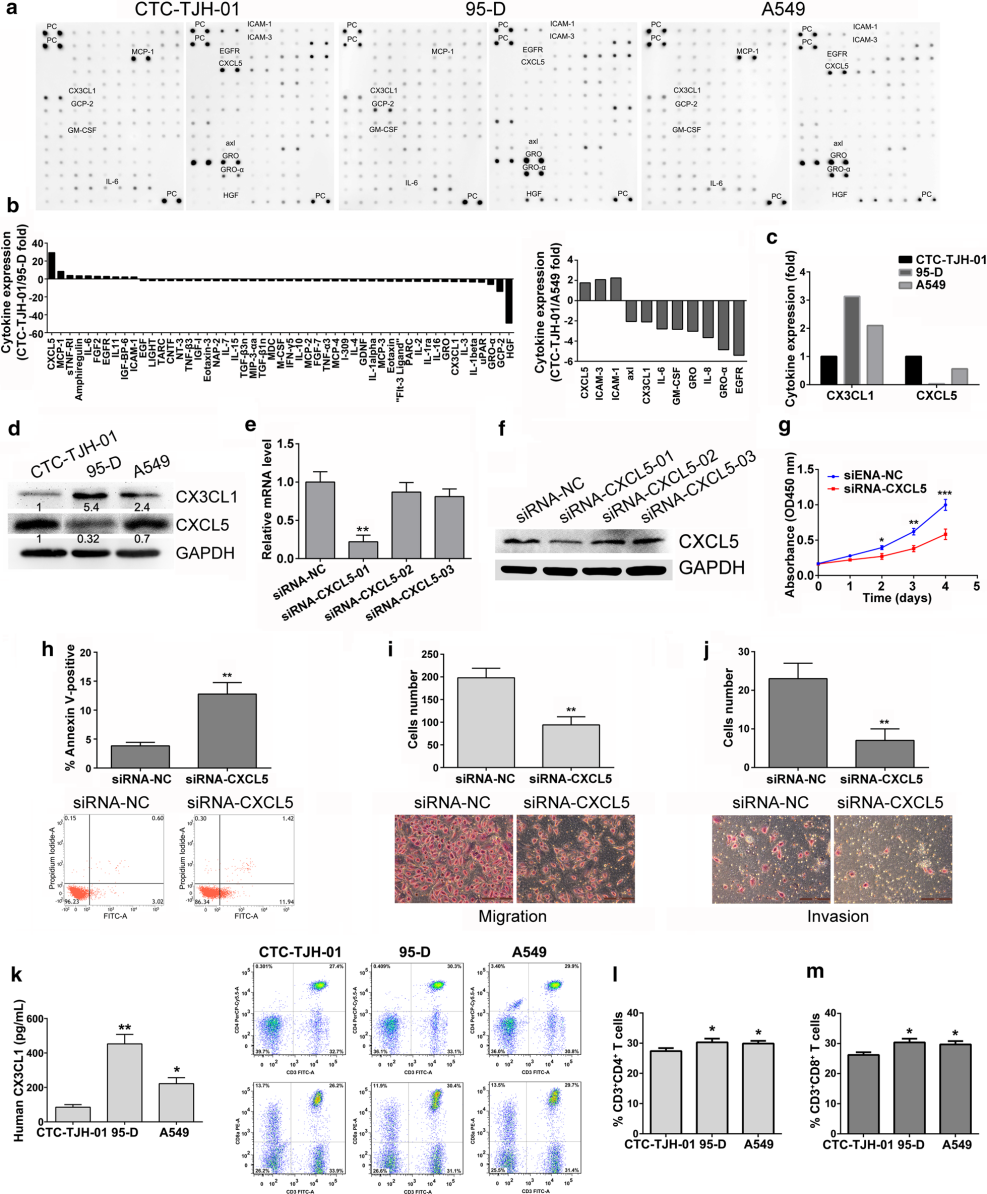
Cytokine chip analysis delineates a different cytokine production signature of CTCs. **a** The cytokine profile array from the cell culture supernatants of the CTC-TJH-01, 95-D and A549 cells were detected by capture antibodies spotted in duplicate on nitrocellulose membranes. **b** The quantitative analysis of cytokine profile in A. **c** The fold changes of CX3CL1 and CXCL5 in the CTC-TJH-01, 95-D and A549 cells. **d** Verification of CX3CL1 and CXCL5 expression in the CTC-TJH-01, 95-D and A549 cells by western blot. **e** Real-time PCR and **f** western blotting were conducted to examine the mRNA and protein expression of CXCL5 in the CTC-TJH-01 cells transfected with the CXCL5-specific siRNA or a non-specific siRNA as the negative control (NC), respectively. **g** CCK-8 assay and **h** cell apoptosis assay were used to determine cell proliferation and apoptosis. **i**, **j** A transwell assay was used to determine cell migration and invasion. **k** ELISA assay was used to determine the CX3CL1 secretion level of cell supernatant. **l**, **m** After co-culture of CTC-TJH-01, 95-D and A549 cells with lymphocytes; flow cytometry was used to detect lymphocyte recruitment. Each bar represents the mean ± SD of three separate experiments. *P < 0.05; **P < 0.01; ***P < 0.001

To study the mechanism by which CTCs escape immune killing and metastasize in peripheral blood, we used cytokine antibody arrays to analyze cytokine secretions in the CTC-TJH-01, 95-D and A549 cell culture supernatants. The results showed that compared with 95-D and A549 cells, CTC-TJH-01 cells have a low expression level of lymphocyte recruitment-associated cytokine CX3CL1 and a high expression level of metastasis-associated protein CXCL5 (Fig. 3a–d). In addition, siRNA interference assay showed that down-regulation of CXCL5 protein significantly inhibited proliferation, invasion and metastasis of CTC-TJH-01 cells (Fig. 3e–j). More importantly, the recruitment of T lymphocytes by 95-D and A549 cells was significantly stronger than that of CTC-TJH-01 cells, probably due to the secretion of higher levels of CX3CL1 factor (Fig. 3k–m). The above results may partly explain the mechanism of CTC metastasis.

### CTCs xenograft has tumorigenicity in immunodeficient mice

**Fig. 4:**
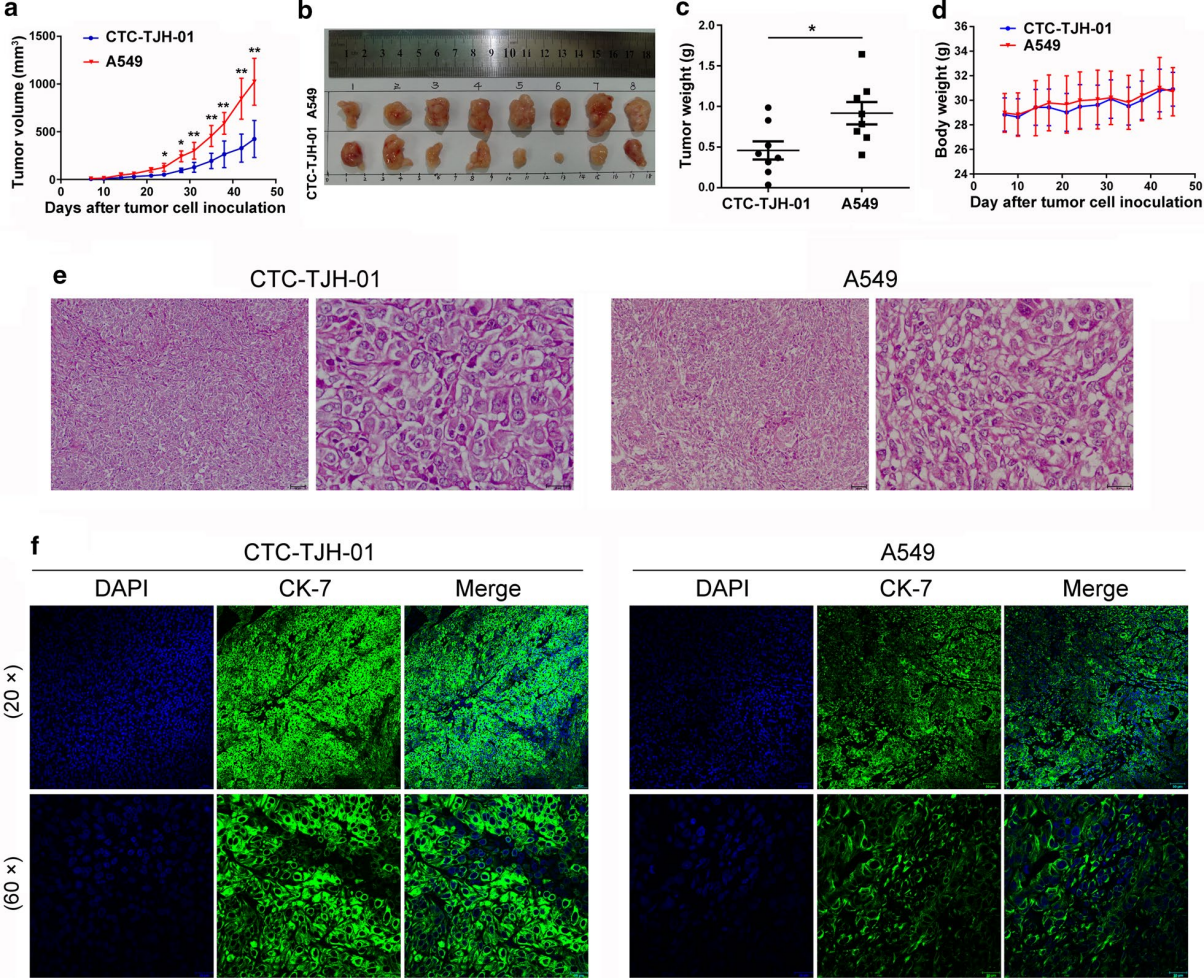
Subcutaneous CTC xenograft tumors exhibits tumorigenicity. **a** The growth curves of the tumors arising from the inoculation with CTC-TJH-01 or A549 tumor cells. Tumor growth was measured with a digital caliper. **b** A representative picture of the excised CTC-TJH-01 and A549 tumors from different mice on the 45th day after implantation. **c** Tumor weight was measured with an electronic balance. **d** Mice were weighed two times a week. **e** H&E staining of CTC-TJH-01 and A549 cell xenografts. Scale bar, 20 and 50 μm. **f** Immunofluorescence analyses of CK-7 expression levels in the CTC-TJH-01 or A549 tumor tissues. Scale bar, 20 and 50 μm. Each bar represents the mean ± SD of three separate experiments. *P < 0.05; **P < 0.01; ***P < 0.001

To study whether CTC-TJH-01 cells have tumorigenicity, we established a circulating tumor cells derived xenograft (CDX) model in immunodeficient mice. The results showed that the CTC-TJH-01 cells had tumorigenicity in NOD/SCID mice. The tumors derived from the A549 cells were significantly larger and had a significantly higher growth rate compared with those from the CTC-TJH-01 cells, which are consistent with in vitro results (Fig. 4a–c). We also found that the growth of CTC-TJH-01 and A549 cells did not significantly affect the weight of NOD/SCID mice (Fig. 4d). In addition, H&E staining confirmed that the CTC-TJH-01 cells had large nuclei and a large karyoplasm (Fig. 4e), and immunofluorescence results showed that CTC-TJH-01 cells have a high CK7 expression level (Fig. 4f). These results indicate that CTC-TJH-01 cells can be used to establish CDX models.

### CTCs xenograft has lower immunostimulation

**Fig. 5:**
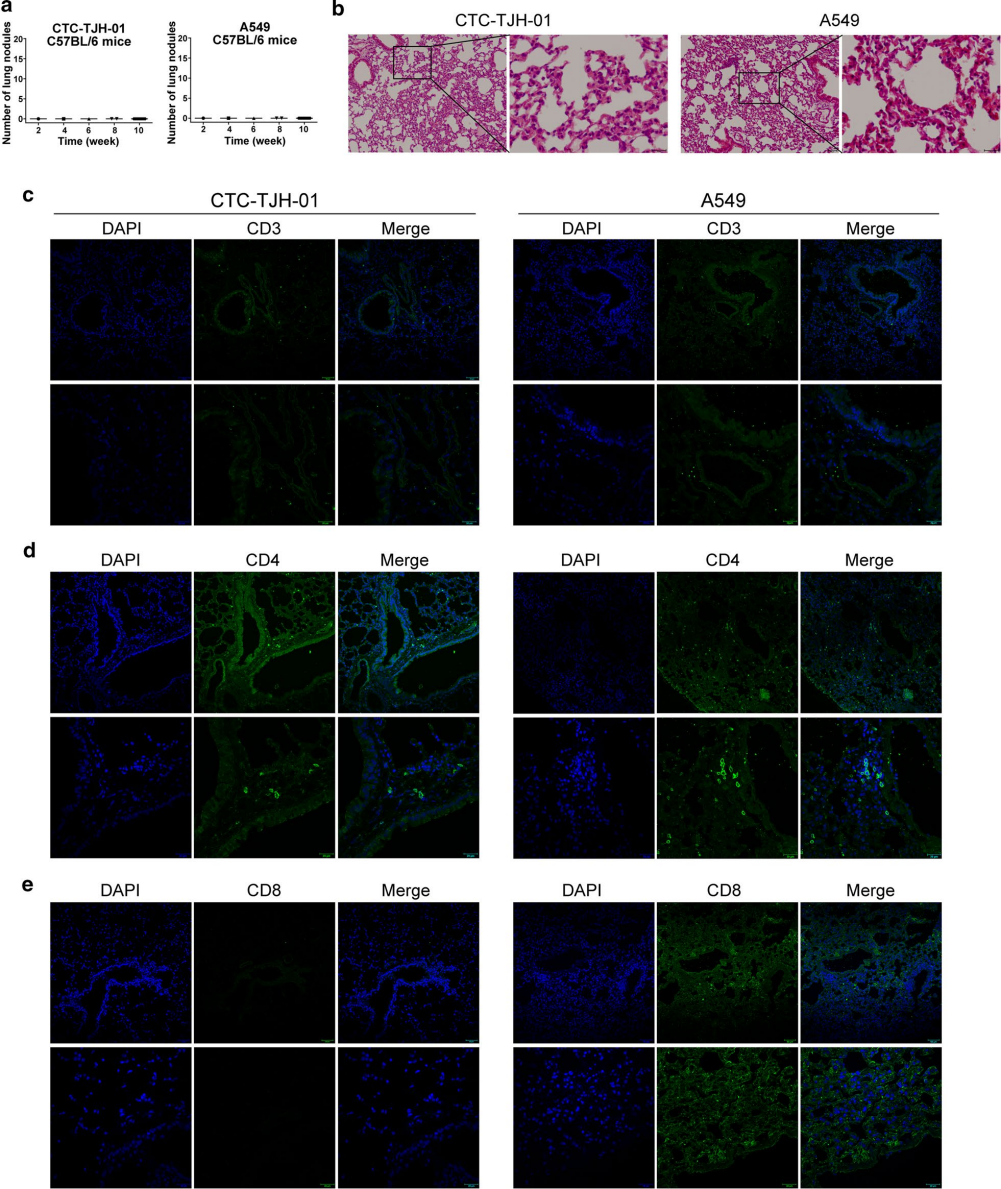
CTCs xenograft has lower immunostimulation. **a** Quantification of lung metastatic foci of C57BL/6 mice (n = 18) after CTC-TJH-01 or A549 cells tail vein inoculation. **b** H&E-stained lung sections of C57BL/6 mice after CTC-TJH-01 or A549 cells xenografts. **c** Immunofluorescence analyses of CD3 expression levels in the CTC-TJH-01 or A549 lung tissues. **d** Immunofluorescence analyses of CD4 expression levels in the CTC-TJH-01 or A549 lung tissues. **e** Immunofluorescence analyses of CD8 expression levels in the CTC-TJH-01 or A549 lung tissues. Scale bar, 20 and 50 μm

To investigate CTC-TJH-01 cells with low expression of CX3CL1 protein may have lower immunostimulation. We used C57BL/6 mice to inoculate CTC-TJH-01 and A549 cells by tail vein injection. Because of immunological rejection, CTC-TJH-01 and A549 cells did not grow in the lungs of C57BL/6 mice (Fig. 5a). In addition, there was no significant difference in lung pathology and expression of CD3^+^ and CD4^+^ T cells between the two groups of mice (Fig. 5b–d). However, A549 cells significantly caused recruitment of CD8^+^ T lymphocytes in the lungs of C57BL/6 mice when compared to CTC-TJH-01 cells (Fig. 5e). These results shown that CTC-TJH-01 cells has low immunostimulation.

### CTCs xenograft has metastatic and organ colonization abilities

**Fig. 6:**
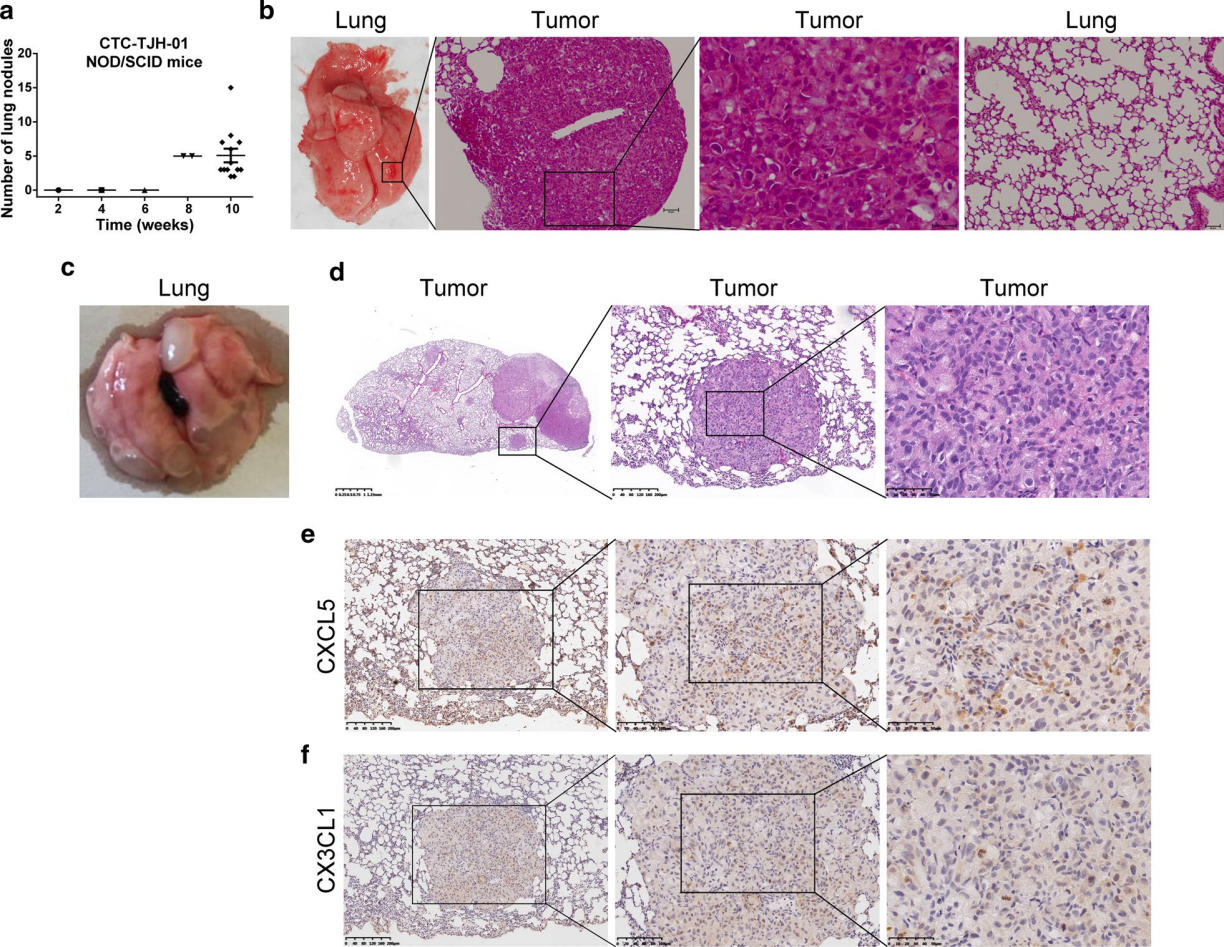
CTCs xenografts has metastatic and organ colonization abilities. **a** Quantification of lung metastatic foci of NOD/SCID mice (n = 18) after CTC-TJH-01 tumor inoculation. **b** A representative picture of lung metastatic foci and H&E-stained after CTC-TJH-01 tumor tail vein inoculation. **c** A representative picture of lung metastatic foci after CTC-TJH-01 tumor subcutaneous inoculation. **d** A representative picture of H&E-stained lung sections of NOD/SCID mice (n = 10) after CTC-TJH-01 tumor subcutaneous inoculation. **e** Immunohistochemical analysis of CXCL5 expression levels in the CTC-TJH-01 tumor tissues. **f** Immunohistochemical analysis of CX3CL1 expression levels in the CTC-TJH-01 tumor tissues

In NOD/SCID mice, we also studied the lung organ colonization ability of CTC-TJH-01 cells by tail vein injection. At 8 weeks after inoculation, metastatic lesions were observed for the first time. At 10 weeks, all remaining mice were sacrificed, and the lung metastases were counted. The average number of lung metastases per mouse was five (Fig. 6a). H&E stained results showed that the density of the lung metastases was higher than that of the subcutaneous xenograft tumor (Fig. 6b). In addition, we also found that the CTC-TJH-01 cells could metastasize from subcutaneous tumor to the lung (Fig. 6c). The pathological results of CTC-TJH-01 cells were confirmed by H&E staining (Fig. 6d). Moreover, immunohistochemical results showed that CTC-TJH-01 cells have a high expression level of CXCL5 and a low expression level of CX3CL1 (Fig. 6e, f). Our integrative study suggests that the expression of CXCL5 and CX3CL1 on CTC may be crucial for its metastasis.

## Discussion

As metastasis is a key factor in determining the death of lung cancer patients, preventing the occurrence of metastasis could prolong the survival of such patients [18]. NCCN guidelines for early stage lung cancer patients include chemotherapy, but only 5.4–6.9% patients showed a benefit from routine treatment, as shown by 5-year survival rates [19]. Clinical studies using targeted therapy and immunotherapy to prevent metastasis in early stage lung cancer patients all failed [20–22]. The main reason was that the interventions were designed to interfere with the proliferation of primary cancer cell, while CTCs, the key player in metastasis, were ignored.

In this study, we have shown that CTC counts were significantly higher in lung cancer patients with early stage than with late stage disease. This finding differs from the majority of clinical reports [23], but is in line with a previous study by Olmedo et al. [24]. The reason may be that we use epithelial markers for enrichment detection, however, after treatment of lung cancer patients, the tumor phenotype of epithelial will be transformed into mesenchymal, which leads to less CTCs in patients with advanced lung cancer. Furthermore, our study patients had different treatments and our CTCs detection method differed from those of other researchers. These results highlight the high risk of metastasis in early postoperative lung cancer patients and demonstrate that CTCs may be a key target for intervention.

We used immunomagnetic beads combined with a microfluidic chip technology to isolate the CTCs and perform ex vivo culture, and only the cells from two lung adenocarcinoma patients were successfully cultured (a Stage IIa patient and a Stage IV patient). However, one of them survived only 6 months, and the final success of this case is from Stage IIa patients. We discovered the proliferation and metastatic ability of CTC-TJH-01 cells is weak, which may be one of the reasons for the unsuccessful culturing of the CTC in vitro. In addition, our study revealed that CTC-TJH-01 cells are more resistant to cisplatin and taxotere compared with A549 and 95-D cells. Yu and his colleagues also found that CTCs from breast cancer are sensitive to paclitaxel and capecitabine and resistant to fulvestrant, doxorubicin, and olaparib [7].

To confirm that our CTC cell line originated from the primary lesion, we previous studies have demonstrated that the sequencing of CTC-TJH-01 cells exhibits wild-type EGFR and a missense mutation at codon 12 in exon 2 of KRAS, which is consistent with the mutation status found in the primary tumor [25]. Moreover, we show that CTC-TJH-01 cells have high expression levels of EMT conversion related proteins E-cadherin, N-cadherin, and high expression levels of stem cell marker proteins CD44 and ALDH1. CTC-TJH-01 was shown (i) an intermediate epithelial/mesenchymal phenotype, (ii) stem cell-like characteristics, and (iii) immune escape characteristics. Laure and colleagues also found similar results in human circulating colon cancer cells [8].

Surprisingly, the levels of cytokines secreted by the CTC-TJH-01 cells were significantly different from those of the 95-D and A549 cells. Our findings suggest that CTC-TJH-01 cells have a low expression level of lymphocyte recruitment factor fractalkine (CX3CL1) and a high expression level of metastasis associated factor CXCL5. Previous research has found that high expression levels of CX3CL1 in tumor cells can induce tumor-infiltrating CD8^+^ T cells, dendritic cells, and activated natural killer cells to exert antitumor immune activity, thus inhibiting tumor growth [26, 27]. Our study found that CTC-TJH-01 cells with low expression of CX3CL1 have weaker recruitment ability for T lymphocytes, suggesting that their immunogenicity is lower. Clinical research has also found that a high CXCL5 expression level is associated with short overall survival and progression-free survival in lung cancer patients [28]. CXCL5 can promote the proliferation and metastasis of lung cancer cells [29]. We show evidence that when CXCL5 expression was down-regulated on CTC-TJH-01 cells, its invasion and migration was significantly inhibited.

Most importantly, to further investigate the tumorigenicity of the CTC-TJH-01 cell line, we established circulating tumor cell-derived xenograft (CDX) models. The rate of CTC tumor formation was 100% in NOD/SCID mice. Recent studies have found that circulating tumor cells from patients with breast, colorectal and small cell lung cancer can also form tumors in immunodeficient mice [7, 8, 10]. In addition, we found that CTC had a lower immunostimulus, possibly due to its low expression of lymphocyte recruitment-related factor CX3CL1. Besides, our innovative discovery those CTC-TJH-01 cells can not only organ colonization to the lung, but also have the ability to metastasize. The above studies indicate that CTCs from the peripheral blood of early lung cancer patients have the ability to metastasize, and the intermediate stage of EMT transformation may be more conducive to metastasis. A recent study identified EMT and its intermediate states as the crucial drivers of tumor progression [30].

## Conclusions

In summary, we established a circulating lung tumor cell line, CTC-TJH-01, that was stable in culture for at least 24 months. Our findings revealed that CTC-TJH-01 cells with an intermediate epithelial/mesenchymal phenotype, stem cell-like characteristics, and immune escape characteristics. Besides, CTC-TJH-01 xenograft has metastatic and lung organ colonization abilities. The high expression of CXCL5 protein and low expression of CX3CL1 protein may be the mechanism of CTC-TJH-01 cell escaping from immune killing and metastasis. Meanwhile, our also finding that the CTC count is greater in early stage NSCLC patients than in late stage patients. These findings suggest that CTCs in peripheral blood of patients with early lung cancer after surgery has the ability to evade immune killing and thus to develop distant metastasis.

## Abbreviations

CTC: circulating tumor cell
NSCLC: non-small cell lung cancer
H&E stain: hematoxylin and eosin stain
EMT: epithelial–mesenchymal transition
EGF: epidermal growth factor
FGF2: fibroblast growth factor 2
TCM: Traditional Chinese Medicine
CCK-8: cell counting kit-8
NCCN: National Comprehensive Cancer Network
EpCAM: epithelial cell adhesion molecule
EGFR: epidermal growth factor receptor
CX3CL1: C-X3-C motif chemokine ligand 1
CXCL5: C-X-C motif chemokine ligand 5
CDX models: circulating tumor cell-derived xenograft models
PBMC: peripheral blood mononuclear cells

## References

[1] Bray F, Ferlay J, Soerjomataram I, Siegel RL, Torre LA, Jemal A (2018). Global cancer statistics 2018: GLOBOCAN estimates of incidence and mortality worldwide for 36 cancers in 185 countries. CA Cancer J Clin.

[2] Massague J, Obenauf AC (2016). Metastatic colonization by circulating tumour cells. Nature.

[3] Alama A, Truini A, Coco S, Genova C, Grossi F (2014). Prognostic and predictive relevance of circulating tumor cells in patients with non-small-cell lung cancer. Drug Discov Today.

[4] Tognela A, Spring KJ, Becker T, Caixeiro NJ, Bray VJ, Yip PY, Chua W, Lim SH, de Souza P (2015). Predictive and prognostic value of circulating tumor cell detection in lung cancer: a clinician’s perspective. Crit Rev Oncol Hematol.

[5] Cheng M, Liu L, Yang HS, Liu GF (2014). Circulating tumor cells are associated with bone metastasis of lung cancer. Asian Pac J Cancer Prev.

[6] Alix-Panabieres C, Pantel K (2014). Challenges in circulating tumour cell research. Nat Rev Cancer.

[7] Yu M, Bardia A, Aceto N, Bersani F, Madden MW, Donaldson MC, Desai R, Zhu H, Comaills V, Zheng Z (2014). Cancer therapy. Ex vivo culture of circulating breast tumor cells for individualized testing of drug susceptibility. Science.

[8] Cayrefourcq L, Mazard T, Joosse S, Solassol J, Ramos J, Assenat E, Schumacher U, Costes V, Maudelonde T, Pantel K (2015). Establishment and characterization of a cell line from human circulating colon cancer cells. Cancer Res.

[9] Sheng W, Ogunwobi OO, Chen T, Zhang J, George TJ, Liu C, Fan ZH (2014). Capture, release and culture of circulating tumor cells from pancreatic cancer patients using an enhanced mixing chip. Lab Chip.

[10] Hodgkinson CL, Morrow CJ, Li Y, Metcalf RL, Rothwell DG, Trapani F, Polanski R, Burt DJ, Simpson KL, Morris K (2014). Tumorigenicity and genetic profiling of circulating tumor cells in small-cell lung cancer. Nat Med.

[11] Zhang L, Ridgway LD, Wetzel MD, Ngo J, Yin W, Kumar D, Goodman JC, Groves MD, Marchetti D (2013). The identification and characterization of breast cancer CTCs competent for brain metastasis. Sci Transl Med.

[12] Gao D, Vela I, Sboner A, Iaquinta PJ, Karthaus WR, Gopalan A, Dowling C, Wanjala JN, Undvall EA, Arora VK (2014). Organoid cultures derived from patients with advanced prostate cancer. Cell.

[13] Jiang R, Lu YT, Ho H, Li B, Chen JF, Lin M, Li F, Wu K, Wu H, Lichterman J (2015). A comparison of isolated circulating tumor cells and tissue biopsies using whole-genome sequencing in prostate cancer. Oncotarget.

[14] Scholch S, Garcia SA, Iwata N, Niemietz T, Betzler AM, Nanduri LK, Bork U, Kahlert C, Thepkaysone ML, Swiersy A (2016). Circulating tumor cells exhibit stem cell characteristics in an orthotopic mouse model of colorectal cancer. Oncotarget.

[15] Wang Z, Wu W, Wang Z, Tang Y, Deng Y, Xu L, Tian J, Shi Q (2016). Ex vivo expansion of circulating lung tumor cells based on one-step microfluidics-based immunomagnetic isolation. Analyst.

[16] Li Q, Liang X, Wang Y, Meng X, Xu Y, Cai S, Wang Z, Liu J, Cai G (2016). miR-139-5p inhibits the epithelial–mesenchymal transition and enhances the chemotherapeutic sensitivity of colorectal cancer cells by downregulating BCL2. Sci Rep.

[17] Li H, Yang T, Ning Q, Shang D, Yao Y, Sun Z (2017). WITHDRAWN: microRNA-505 modulates cancer proliferation and migration in human non-small cell lung cancer through inverse regulation of FZD4. Lung Cancer..

[18] Rusch VW, Chansky K, Kindler HL, Nowak AK, Pass HI, Rice DC, Shemanski L, Galateau-Salle F, McCaughan BC, Nakano T (2016). The IASLC Mesothelioma Staging Project: proposals for the M descriptors and for revision of the TNM stage groupings in the forthcoming (Eighth) edition of the TNM classification for mesothelioma. J Thorac Oncol.

[19] Pignon JP, Tribodet H, Scagliotti GV, Douillard JY, Shepherd FA, Stephens RJ, Dunant A, Torri V, Rosell R, Seymour L (2008). Lung adjuvant cisplatin evaluation: a pooled analysis by the LACE Collaborative Group. J Clin Oncol.

[20] Vansteenkiste JF, Cho BC, Vanakesa T, De Pas T, Zielinski M, Kim MS, Jassem J, Yoshimura M, Dahabreh J, Nakayama H (2016). Efficacy of the MAGE-A3 cancer immunotherapeutic as adjuvant therapy in patients with resected MAGE-A3-positive non-small-cell lung cancer (MAGRIT): a randomised, double-blind, placebo-controlled, phase 3 trial. Lancet Oncol.

[21] Goss GD, O’Callaghan C, Lorimer I, Tsao MS, Masters GA, Jett J, Edelman MJ, Lilenbaum R, Choy H, Khuri F (2013). Gefitinib versus placebo in completely resected non-small-cell lung cancer: results of the NCIC CTG BR19 study. J Clin Oncol.

[22] Uemura T, Hida T (2018). Durvalumab showed long and durable effects after chemoradiotherapy in stage III non-small cell lung cancer: results of the PACIFIC study. J Thorac Dis.

[23] Wu C, Hao H, Li L, Zhou X, Guo Z, Zhang L, Zhang X, Zhong W, Guo H, Bremner RM (2009). Preliminary investigation of the clinical significance of detecting circulating tumor cells enriched from lung cancer patients. J Thorac Oncol.

[24] Olmedo ME, Mezquita L, Earl J, Benito A, Santon A, Longo F, Vallejo C, Muñoz G, Gorospe L, Soria A (2014). 248P monitoring circulating tumor cells (CTC) in lung cancer: preliminary results. Ann Oncol.

[25] Zhang Y, Tang Y, Sun S, Wang Z, Wu W, Zhao X, Czajkowsky DM, Li Y, Tian J, Xu L (2015). Single-cell codetection of metabolic activity, intracellular functional proteins, and genetic mutations from rare circulating tumor cells. Anal Chem.

[26] Siddiqui I, Erreni M, van Brakel M, Debets R, Allavena P (2016). Enhanced recruitment of genetically modified CX3CR1-positive human T cells into Fractalkine/CX3CL1 expressing tumors: importance of the chemokine gradient. J Immunother Cancer.

[27] Park MH, Lee JS, Yoon JH (2012). High expression of CX3CL1 by tumor cells correlates with a good prognosis and increased tumor-infiltrating CD8^+^ T cells, natural killer cells, and dendritic cells in breast carcinoma. J Surg Oncol.

[28] Wu K, Yu S, Liu Q, Bai X, Zheng X, Wu K (2017). The clinical significance of CXCL5 in non-small cell lung cancer. Onco Targets Ther.

[29] Wang L, Shi L, Gu J, Zhan C, Xi J, Ding J, Ge D (2018). CXCL5 regulation of proliferation and migration in human non-small cell lung cancer cells. J Physiol Biochem.

[30] Nieto MA, Huang RY, Jackson RA, Thiery JP (2016). Emt: 2016. Cell.

